# Genome-wide and transcriptome analysis of PdWRKY transcription factors in date palm (*Phoenix dactylifera*) revealing insights into heat and drought stress tolerance

**DOI:** 10.1186/s12864-025-11715-6

**Published:** 2025-07-01

**Authors:** Ibrahim Khan, Lubna, Saqib Bilal, Ashraf M. M. Abdelbacki, Sang-Mo Kang, Ahmed AL-Harrasi, Sajjad Asaf, In-Jung Lee

**Affiliations:** 1 Natural and Medical Science Research Center, University of Nizwa, 616 Nizwa, Oman; 2 Department of Applied Biosciences, Kyungpook National University, Daegu, 41566 Republic of Korea; 3 Department of Botany and Microbiology, College of Science, King Saud University, P.O. Box 2455, 11451 Riyadh, Saudi Arabia

**Keywords:** *Phoenix dactylifera*, TFs, *PdWRKY*, Drought, Heat, Phylogenetic analysis, Gene ontology, KEGG

## Abstract

Genomic and transcriptomic studies on date palm (*Phoenix dactylifera*) are still inadequate, but several studies have contributed to understanding its genetic makeup, especially in the Khalas cultivar. Looking at the extensive importance of *WRKY* transcription factors (TFs) in plant growth, development and defense responses to various biotic and abiotic stresses, we conducted a study to identify and functionally annotate the *WRKY* TFs of *P. dactylifera*, with a particular focus on their involvement in drought and heat stress. A total of 73 *PdWRKY* TFs were curated and classified into 7 and 17 clades through comparative phylogenetic analysis and orthologous comparison of the *WRKY* TFs from the extensively studied genomes of *Arabidopsis thaliana* and *Oryza sativa*, respectively. Our findings show that 52% of *PdWRKYs* have strong homology with *OsWRKYs*, while only 9 *PdWRKYs* have orthologous relationships with *AtWRKYs*. This indicates a divergence in evolutionary patterns, likely due to gene duplications and losses in rice, *Arabidopsis*, and date palm, that occurred both before and after the last common ancestor of these species. Our comprehensive analysis of gene structures, conserved motifs, and protein-protein interactions confirmed functional similarities among many *PdWRKYs*. The GO and KEGG pathway enrichment analyses validate that *PdWRKY* genes have significant functional roles in various molecular, cellular, and biological processes. The transcriptomics analysis revealed that heat stress resulted in upregulating 7 genes (12.2%) and downregulating 10 *PdWRKY* genes (17.5%). With combined drought and heat stress, 15 genes (26.3%) were upregulated, and 9 genes (15.78%) were downregulated. Notably *PdWRKYs* genes such as LOC103723396 (*WRKY35*) and LOC103718774 (*WRKY1*) showed significantly higher while LOC103713231(*WRKY28*), LOC103721327(*WRKY72*, LOC103721580 (*WRKY50*), LOC120110335 (*WRKY70*), and LOC103707788 (*WRKY71*) showed significantly lower expression under drought and heat stress conditions compared to control plants, indicating their vital role in adaptation and tolerance mechanisms against these environmental stresses. The current study will highlight the essential role of comprehensive genomic and transcriptomic studies for developing innovative approaches to promote plant growth in adverse conditions. Further studies on these *PdWRKY* genes could provide insights into their specific roles and mechanisms in stress response pathways in date palms, potentially leading to strategies for improving stress resilience in this important crop species.

**Supplementary Information:**

The online version contains supplementary material available at 10.1186/s12864-025-11715-6.

## Introduction

Date palm (*Phoenix dactylifera* L.) is a perennial, monocotyledonous, and dioecious fruit tree belonging to the Arecaceae family. The Arecaceae, also known as the palm family, include around 2,500 species and over 200 genera [1]. Date palm cultivation has its historical and geographical origins primarily in the arid regions of the Middle East, North Africa, and the Arabian Peninsula, where it has grown successfully for thousands of years, adopting to extreme heat and limited water availability [2, 3]. Dates production in the Arabian Peninsula accounts for approximately 34% of global output, with Saudi Arabia, Iraq, the United Arab Emirates, and Oman being the leading producers in the region [4]. Its importance in these regions extends beyond food production, as date palms are integral to environmental conservation. They help combat desertification, reduce soil erosion, and enhance water retention, thus playing a crucial role in stabilizing fragile ecosystems [5]. In addition to their environmental benefits, date palms have significant nutritional, economic, and aesthetic benefits. Date fruits are a cost-effective source of essential micro and macronutrients [6, 7]. They are primarily composed of carbohydrates, including soluble sugars and dietary fiber, with minimal quantities of fats and proteins [8, 9]. In addition to these macronutrients, dates are a good source of various vitamins (B3, B5, B6 and B9) and minerals including vitamin, copper, sulfur, iron, magnesium and potassium [8, 10, 11]. Date fruits are also known for their important nutraceuticals, which provide a wide range of benefits including anti-mutagenic, anti-inflammatory, antioxidant, antibacterial, hepatoprotective, gastroprotective, and immune-stimulatory activities [6, 12]. Despite the immense importance of the date palm in terms of nutrition, environment, and economy, comprehensive genomic and transcriptomic studies on this crop are still inadequate [13]. Date palm is an interesting candidate for studying drought tolerance and exploring its potential for improving agricultural resilience. Its natural ability to thrive in harsh, water-scarce environments makes it a valuable resource for understanding the genetic and physiological mechanisms associated with drought resistance [14, 15]. Conversely, development of improved varieties is required to combat various biotic and abiotic stresses that affect crop cultivation. The genetic map construction for the “Khalas cultivar” was carried out in 2015 [16]. Prior to this, various genomic studies were conducted, including sequencing of the chloroplast [17, 18] mitochondria [19] and the whole nuclear genome [20, 21]. These investigations have contributed to a comprehensive understanding of the genetic makeup and structure of date palms, especially the Khalas cultivar. However, the key genes involved in the tolerance mechanism remain unknown [22]. Investigating TF families is an important aspect of the post-genomic era [23]. TFs are proteins that bind to particular DNA sequences and regulate the transcription of genetic information from DNA to RNA in order to regulate the expression of genes [24]. A typical TF is made up of two domains: the DNA-binding domain (DBD) and the functional domain. The DBD allows the TF to bind to specific DNA sequences, whereas the functional domain regulates transcriptional activation and/or repression in response to endogenous and exogenous stimuli. Ultimately, TFs play a crucial role in mediating various physiological and biochemical processes. Finally, TFs serve an important function in regulating a variety of physiological and biochemical processes [25, 26]. They significantly influence the transcriptional regulation of genes associated with immunological responses, cell cycle regulation, growth, development, differentiation, and environmental stress responses [27, 28]. Like many other organisms date palm dedicates approximately 7% of its all expressed genes to encode TFs [29]. Based on their unique DNA-binding domains, TFs are categorized into different gene families [30]. Understanding TFs and their roles in date palm is crucial for advancing agricultural practices, enhancing crop yield, improving stress tolerance, and overall plant development. However, still there are no comprehensive reports available on the genome and transcriptome-wise study of date palm TFs. Among them, *WRKY* TFs are the most important and widely distributed in plants. In plant biology, *WRKY* TFs are often referred to as 'jacks-of-all-trades' due to their extensive involvement in multiple physiological and developmental processes, including responses to various biotic and abiotic stress stimuli [24, 31, 32]. The *WRKY* domain is typically composed of a characteristic peptide sequence of 60-70 amino acid residues. This domain contains highly conserved amino acid motifs, notably WRKYGQK or WRKYGKK, which are vital for DNA binding to certain promoter regions known as W-Box [33, 34]. These promoter elements have a recognition sequence at the N-terminus of (T)(T)TGAC(C/T), and at the C-terminus, they contain a zinc finger motif of either the C_2_H_2_ or C_2_HC type [24, 35]. Extensive studies on *WRKY* TFs across various plant species has provided insights into their essential roles in responding to drought and heat stress [36, 37]. In *Arabidopsis*, 72 *WRKY* TFs have been curated in our current study. It has been demonstrated that overexpression of *AtWRKY11* and *AtWRKY17* improves drought tolerance by promoting seed germination and root growth under drought-stressed conditions [38]. *AtWRKY25* and *AtWRKY33* are recognized for their involvement in responses to heat and osmotic stresses [39]. Similarly, the current study systematically identified 101 *OsWRKY* TFs in rice. Among these, *OsWRKY11* is involved in upregulating the expression of HSP101, which significantly enhances the plant's resistance to both drought and heat stress [40]. *WRKY* TFs generally mediate drought stress responses via the abscisic acid (ABA) signaling pathway [41]. *AtWRKY57* improves drought stress tolerance in *Arabidopsis* by binding to W-box elements and activating the expression of *RD29A* and *NCED3* [42]. *TaWRKY2* and *TaWRKY19* bind to the W-box elements of stress responsive genes, such as *STZ*, *RD29B*, *DREB2A*, and *Cor6.6*, significantly enhancing drought and salt tolerance in wheat [43]. *GmWRKY54* in soybean improves resistance to drought stress by binding to the promoter regions of *PYL8*, *CPK3*, *CIPK1*, and *SRK2A* and activating their expression [44]. Similarly, *WRKY53* in *Pyrus betulaefolia* improves tolerance to drought stress by interacting with the W-box element of *PbrNCED1* [45].

Recently meta-omics approaches including genomics, transcriptomics, proteomics, and metabolomics are continually evolving, and research on specific TFs and their functions is continuously expanding [46, 47]. Genome- and transcriptome-wide studies are essential for advancing our understanding of plant physiology and evolution. These studies significantly improve crop productivity and resilience and contribute to the preservation of biodiversity and addressing global challenges related to food security and environmental sustainability [48–50]. In the current study, we focused on comparative genomic and transcriptome studies, as well as functional characterization of *WRKY* TFs in *P. dactylifera*, with the rationale being its recently sequenced whole genome and its ecological and economic significance [21]. Our primary objectives were to identify and functionally characterize the *WRKY* TFs family in the *P. dactylifera* genome by employing various *in-silico* approaches, including phylogenetics, gene architecture analysis, conserved motif identification, protein-protein interactions, gene ontology, co-expression patterns, and expression profiling. Our findings reveal detailed insights into the WRKY TFs family in *P. dactylifera*, providing a foundation for understanding their roles in heat and drought stress responses.

## Materials and methods

### Identification of PdWRKY protein

Coding DNA (CDS) and deduced amino acid sequences of *PdWRKY* were retrieved from the Plant Transcription Factor Database v5.0 (PlantTFDB 5.0; https://www.planttfdb.gao-lab.org/ and the iTAK-Plant Transcription factor & Protein Kinase Identifier and Classifier (http://www.itak.feilab.net/cgi-http://www.bin/itak/db_browse.cgi). Subsequently, the protein sequences of *AtWRKY* and *EgWRKY* were retrieved from the Arabidopsis Information Resource (TAIR, https://www.arabidopsis.org/browse/gene_family/WRKY) and the National Center for Biotechnology Information (NCBI, https://www.ncbi.nlm.nih.gov/), respectively. The genomic sequences were downloaded by searching for iTAK accession numbers on NCBI, identified the corresponding LOC accession numbers and subsequently obtained the genomic sequences through a BLAST search (Table S1). Expasy ProtParam (https://www.web.expasy.org/protparam) and NCBI (https://www.ncbi.nlm.nih.gov/) were used to determine and analyze the nucleotides length (bp), predicted proteins length (aa), molecular weight (Da) and theoretical isoelectric point of the protein sequences of potential *WRKY* members. Additionally, CELLO (http://www.cello.life.nctu.edu.tw/) was used to predict the subcellular locations of the *PdWRKY* proteins (Table 1). The identified *WRKY* TFs were named *PdWRKY* to indicate their origin from *P. dactylifera*.

**Table 1:** The data of 73 PdWRKY genes identified in *P*. *dactylifera* genome

**S.No**	**iTAK Name**	**STRING Name/ Accession**	**Gene Description**	**Chromosome No**	**Chromosomal Location**		**Nucleotide length (bp)**	**Predicted protein length (aa)**	**Predicted molecular weight (Da)**	**Predicted pI**	**Sub-Cellular Location**	**Exon Number**
					**Start**	**End**						
1	PDK_30s1008821g006	LOC103723227	WRKY51-like	Unknown			513	174	19329.75	7.6	Extracellular, nuclear and chloroplast	3
2	PDK_30s1011261g001	LOC103709614	WRKY51-like	9	21,223,814	21,225,222	615	204	22863.67	5.33	Nuclear	3
3	PDK_30s1025361g003	LOC103717512	WRKY24-like	3	4,286,295	4,289,386	1728	575	63083.11	8.28	Nuclear	5
4	PDK_30s1051061g001	LOC103702225	WRKY55-like	3	5,128,421	5,130,908	1251	416	45570.37	5.96	Nulcear and cytoplsmic	2
5	PDK_30s1051061g002	LOC103702224	WRKY70-like	3	5,115,011	5,119,080	852	283	31037.78	5.25	Nulcear and cytoplsmic	3
6	PDK_30s1053181g004	LOC103696297	WRKY44-like	12	6,184,087	6,188,759	1770	589	64856.75	9	Nulcear and mitochondrial	8
7	PDK_30s1054411g002	LOC103723396	WRKY35-like	1	4,284,574	4,287,626	765	254	28459.2	7.14	Extracellular and nuclear	3
8	PDK_30s1065261g003	LOC103696592	WRKY4-like	7	10,556,090	10,566,970	1137	378	41947.54	9.16	Extracellular and nuclear	4
9	PDK_30s1067021g003	LOC103709613	WRKY51-like	9	21,207,521	21,209,025	516	171	18804.14	8.9	Nuclear	3
10	PDK_30s1078511g002	LOC103698294	WRKY35-like	13	8,753,756	8,755,699	558	185	20290.27	5.02	Nuclear	2
11	PDK_30s1093591g002	LOC103708338	WRKY53-like	11	1,460,107	1,462,876	1089	362	40301.73	6.33	Nuclear	3
12	PDK_30s1103751g023	LOC120104087	WRKY47-like	1	40,285,108	40,290,287	1410	469	51015.99	7.7	Nuclear	6
13	PDK_30s1122501g012	LOC103704540	WRKY1-like	Unknown			1080	359	39435.83	9.72	Nuclear, plasmamembrane and extracellular	5
14	PDK_30s1138471g005	LOC103720433	WRKY72-like	11	2,945,908	2,949,138	825	225	25270.79	6.91	Nuclear, Plasmamembrane and extracellular	4
15	PDK_30s1138471g008	LOC103721580	WRKY50-like	11	2,924,914	2,926,462	582	149	15902.4	4.84	Nulcear and chloroplast	3
16	PDK_30s1140701g002	LOC103717201	WRKY4-like	Unknown			1455	484	52724.88	6.45	Nuclear	4
17	PDK_30s1141221g020	LOC103704459	WRKY57-like	Unknown			459	152	16951.07	9.03	Nuclear	6
18	PDK_30s1149101g001	LOC103697157	WRKY28-like	4	12,090,004	12,092,142	1026	341	38153.3	7.28	Nuclear	3
19	PDK_30s1158601g004	LOC103714091	WRKY51-like	7	389,816	392,154	939	312	34092.04	9.65	Nuclear	3
20	PDK_30s1171101g004	LOC103710925	WRKY22-like	8	17,587,157	17,589,371	402	106	12007.5	10.02	Nuclear	2
21	PDK_30s1173121g001	LOC103707788	WRKY71-like	7	10,277,262	10,279,420	699	232	25364.51	8.97	Nuclear	5
22	PDK_30s1175051g002	LOC103699521	WRKY31-like	9	15,932,154	15,935,769	1584	527	56539.99	6.23	Nuclear	6
23	PDK_30s1200411g002	LOC103716572	WRKY1-like	8	12,886,893	12,890,605	1062	353	39359.84	9.97	Nuclear	3
24	PDK_30s1203911g001	LOC103705538	WRKY72-like	9	21,265,107	21,271,252	1113	370	40891.16	7.13	Nuclear	6
25	PDK_30s65509113g003	LOC103710854	WRKY13-like	Unknown			744	247	27560.59	9.6	Nulcear and mitochondrial	3
26	PDK_30s65509418g001	LOC103706248	WRKY70 -like	3	19,274,531	19,276,594	897	298	33296.35	5.29	Extracellular and nuclear	3
27	PDK_30s6550959g005	LOC103720799	WRKY50-like	8	21,849,949	21,851,404	636	211	23178.3	7.5	Extracellular and nuclear	3
28	PDK_30s65509720g004	LOC103698036	WRKY9-like	6	9,217,829	9,219,916	1155	384	43264.34	5.07	Nuclear	5
29	PDK_30s65509724g007	LOC103718774	WRKY1-like	16	3,654,656	3,658,054	276	327	36540.4	9.8	Nuclear	5
30	PDK_30s655861g002	LOC103708865	WRKY24-like	12	5,217,200	5,220,331	1344	447	49124.97	7.69	Extracellular and nuclear	5
31	PDK_30s663661g001	LOC103709750	WRKY13-like	8	21,045,738	21,051,110	732	243	27177.67	9.61	Nuclear	3
32	PDK_30s665281g005	LOC103720653	WRKY13-like	11	3,229,040	3,234,499	558	282	32157.2	7.67	Nuclear and extracellular	3
33	PDK_30s669181g002	LOC103698763	WRKY75-like	Unknown			465	154	17594.09	9.99	Mitochondrial, extracellular and chloroplast	2
34	PDK_30s680531g012	LOC103706394	WRKY14-like	2	5,083,151	5,087,145	909	302	33013.17	9.21	Nuclear and mitochondrial	3
35	PDK_30s681021g002	LOC103703339	WRKY31-like	8	14,850,874	14,853,414	1428	475	51472.21	7.09	Nuclear	6
36	PDK_30s684781g004	LOC103700856	WRKY4-like	16	8,549,561	8,557,782	765	254	28627.58	9.35	Nuclear	4
37	PDK_30s689431g003	LOC103720801	WRKY71-like	8	21,859,444	21,860,857	354	117	13034.83	9.77	Nuclear, extracellular and mitochondrial	3
38	PDK_30s691921g002	LOC103696886	WRKY48-like	13	7,439,682	7,441,248	789	262	28643.4	9.03	Nuclear and plasmamembrane	3
39	PDK_30s692531g003	LOC103697265	WRKY49-like	3	963,038	968,437	984	327	36307.35	5.09	Nuclear	3
40	PDK_30s705791g001	LOC120112317	WRKY51-like	11	2,906,219	2,908,669	528	175	19957.48	8.67	Nuclear	3
41	PDK_30s716811g001	LOC103707065	WRKY51-like	7	5,434,290	5,435,871	690	229	24902.49	10.02	Nuclear and plasmamemebrane	3
42	PDK_30s722152g002	LOC103713231	WRKY28-like	11	11,179,710	11,181,793	975	324	36271.35	8.27	Nuclear	3
43	PDK_30s723051g005	LOC103723223	WRKY50-like	Unknown			618	205	22303.52	5.41	Nuclear and cytoplasmic	3
44	PDK_30s742801g002	LOC103721507	WRKY24-like	13	9,258,081	9,261,073	1752	583	63893.18	6.45	Nuclear	5
45	PDK_30s745031g002	LOC103702950	WRKY41-like	8	18,496,062	18,501,944	1080	359	39969.18	5.49	Nuclear and extracellular	3
46	PDK_30s771211g003	LOC103714938	WRKY SUSIBA2-like	6	11,784,290	11,795,752	1680	559	61637.75	6.92	Nuclear	8
47	PDK_30s777581g001	LOC103713914	WRKY2-like	10	11,623,067	11,676,463	1599	532	58715.76	5.87	Nuclear	6
48	PDK_30s785891g002	LOC103695902	WRKY41-like	4	4,260,793	4,263,346	1077	358	39898.19	5.76	Nuclear	3
49	PDK_30s806661g003	LOC103707403	WRKY4-like	15	1,903,543	1,918,739	894	297	32509.72	6.31	Nuclear	7
50	PDK_30s816181g002	LOC103702223	WRKY70-like	3	5,102,035	5,104,627	921	306	33708.71	5.62	Nuclear	3
51	PDK_30s816181g003	LOC120110335	WRKY70-like	3	5,463,510	5,465,803	870	289	31775.47	6.66	Nuclear and chloroplast	3
52	PDK_30s835131g001	LOC103720275	WRKY75-like	10	8,418,427	8,421,246	549	182	20981.5	9.52	Cytoplasmic and nuclear	2
53	PDK_30s839411g020	LOC103710421	WRKY40-like	7	2,286,053	2,289,643	762	253	28525.02	6.37	Nuclear and extracellular	4
54	PDK_30s839411g021	LOC103710422	WRKY76-like	7	2,276,980	2,278,745	927	289	32175.05	7.66	Nuclear	5
55	PDK_30s839411g024	LOC103710430	WRKY4-like	7	2,205,250	2,216,164	1362	453	50169.52	8.74	Nuclear	5
56	PDK_30s849681g002	LOC103698690	WRKY4-like	1	28,814,889	28,829,154	876	291	32066.72	8.05	Nuclear and extracellular	4
57	PDK_30s853591g002	LOC103721327	WRKY72-like	8	21,705,322	21,705,322	1548	515	55286.18	8.41	Nuclear	6
58	PDK_30s854791g001	LOC103712650	WRKY14-like	12	7,712,687	7,714,976	532	176	19677.79	4.88	Nuclear	3
59	PDK_30s868971g001	LOC103713754	WRKY65-like	9	19,324,657	19,326,223	227	75	8106.1	10.35	Nulcear and mitochondrial	3
60	PDK_30s877681g002	LOC103724141	WRKY72-like	Unknown			1923	640	69154.09	8.41	Nuclear	6
61	PDK_30s880731g006	LOC103702844	WRKY44-like	3	21,731,302	21,736,384	1206	401	43845.12	9.03	Nuclear	6
62	PDK_30s881991g001	LOC103718944	WRKY2-like	Unknown			1527	582	63237.65	6.3	Nuclear	7
63	PDK_30s888801g002	LOC103710681	WRKY2-like	1	13,753,223	13,758,109	2088	695	75983.51	6.82	Nuclear	5
64	PDK_30s904851g001	LOC103699722	WRKY14-like	5	602,647	606,833	720	239	26587.62	9.18	Nulcear and mitochondrial	3
65	PDK_30s912911g001	LOC103704194	WRKY49-like	3	23,095,771	23,098,486	669	222	24977.42	8.51	Nuclear and extracellular	3
66	PDK_30s914091g001	LOC103705233	WRKY4-like	6	7,071,054	7,073,589	981	361	39304.54	6.67	Nuclear and extracellular	7
67	PDK_30s930251g004	LOC103697177	WRKY49-like	12	7,071,054	7,073,589	867	288	32397.4	5.69	Nuclear	3
68	PDK_30s932191g002	LOC103708157	WRKY41-like	9	17,986,913	17,990,629	1095	364	40576.05	7.18	Nuclear	3
69	PDK_30s936891g001	LOC103716221	WRKY71-like	1	29,156,973	29,158,781	948	315	35120.62	8.94	Nuclear	5
70	PDK_30s955201g001	LOC103711983	WRKY2-like	Unknown			1383	460	49792.87	5.57	Nuclear	6
71	PDK_30s969751g001	LOC103721211	WRKY75-like	14	4,183,177	4,187,713	351	116	13662.45	9.75	Cytoplasmic and nuclear	2
72	PDK_30s975231g001	LOC103715016	WRKY31-like	11	10,767,467	10,769,239	1734	577	61900.9	5.92	Nuclear	4
73	PDK_30s669281g004	LOC103716571	WRKY11-like	Unknown			594	197	21291.24	9.76	Nuclear	1

### Alignment of multiple sequences and phylogenetic analysis

To investigate the evolutionary and paralogues relationships within the *P. dactylifera WRKY* TFs family, individual unrooted trees were constructed using the ML technique in MEGA11 software. The Clustal-W progressive alignment method [51], was used to align the deduced protein sequences. Gap opening and gap extension penalties for both pairwise and multiple sequence alignments were set at 15.00 and 6.66, respectively. Subsequently, with all other parameters set to their default settings, bootstrap (BS) values were calculated from 1,000 repetitions to evaluate the resulting phylogeny. Individual phylogenetic tree of the *PdWRKY* proteins was constructed to analyze their phylogenetic and paralogous relationships with each other. Two well-studied and representative plants, *O. sativa* and *A. thaliana*, were selected in order to investigate the orthologous relationships and functional annotations of *PdWRKY* TFs. Additionally, we included *Elaeis guineensis* due to its close evolutionary relationships with *P. dactylifera*. However, the specific functional roles of the majority of *EgWRKY* genes that form orthologous pairs with *PdWRKY* genes have not been thoroughly investigated. Therefore, to uncover their genome-wide phylogenetic relationships and evolutionary patterns, we aligned the full-length amino acid sequences of the *PdWRKY* genes with *WRKY* TFs of *O. sativa*, *A. thaliana*, and *E. guineensis* and unrooted combined phylogenetic trees were constructed using MEGA software. The resulting phylogenetic trees were visualized and annotated using FigTree software v1.4.4 (http://tree.bio.ed.ac.uk/software/figtree/) [52].

### Exon-intron structural architecture and motif analysis

The Gene Structure Display Server (GSDS 2.0, http://gsds.gao-lab.org/, [53], accessed on 20 February 2024) was used to illustrate the exon-intron architecture of individual *PdWRKY* genes. This analysis involved aligning the CDS sequences with their corresponding genomic sequences. The deduced amino acids sequences of *PdWRKYs* were submitted to and analyzed using the Multiple EM for Motif Elicitation (MEME 5.4.1, https://meme-suite.org/meme/doc/meme.html, accessed on 20 February 2024) [54]. The MEME analysis was performed with a maximum of 15 motifs, allowing any number of repetitions, and the optimum width was set to range ≥6.00 - ≤200 amino acid residues.

### Prediction of protein-protein interaction networks

All putative *PdWRKY* protein sequences in FASTA format were submitted to the web server STRING version 12.0 (https://string-db.org/, accessed February 20, 2024) in order to predict protein-protein interaction networks and functional annotations. The organism was specified as *P. dactylifera* with a high confidence score threshold of 0.700 and the maximum number of interactions was set to 10. Genes with significant confidence scores were utilized in constructing the protein-protein interactions network after blasting. Genes that did not interact with one another were excluded from the network.

### Analysis of cis-acting regulatory elements

To explore cis-acting regulatory elements and their potential roles in regulating stress and light responses, and hormones effects, the 2000 bp upstream sequences designated as promoter regions were retrieved from the NCBI and analyzed using the PlantCARE database (https://bioinformatics.psb.ugent.be/webtools/plantcare/html/) [55].

### Chromosomal localization and gene duplication analysis

Information about the chromosomal localization and length of 61 out of 73 (83.56%) *PdWRKY* genes in the *P. dactylifera* genome was obtained using the BLAST [56] search against the NCBI database [57]. A total of 61 *PdWRKY* genes (83.56%) were successfully mapped to specific locations on sixteen chromosomes on their relative distances using online tools of MG2C version 1.1 (http://mg2c.iask.in/mg2c_v2.1/ accessed on 26 February 2024 [58]. On the other hand, the lengths and chromosomal locations of the remaining 12 (16.43%) *PdWRKY* genes are still unknown. The segmental duplication events occurring in the *PdWRKY* gene family were investigated by conducting a synteny analysis using BLASTP and MCScanX [59] (Table S4). *PdWRKY* genes with aligned sequences showing ≥ 70% similarity across the entire gene length were defined as duplicated genes. In particular, genes that were separated by five or fewer intervening genes and were located within a 100 kb region were considered as tandem duplicates [60].

### Gene ontology-based functional annotation analysis

The online software agriGO version 2.0 (http://systemsbiology.cau.edu.cn/agriGOv2/, accessed on 26 February 2024) with the TopGO “elim” algorithm [61] was used to perform gene ontology (GO) enrichment analysis [62] on *PdWRKY* genes focusing on their involvement in biological processes, molecular functions and cellular components. GO term enrichment was computed using a hypergeometric distribution, applying a P-value cutoff of 0.05 and a term-mapping count cutoff of 5.00. The P-values obtained from Fisher's exact test were subsequently adjusted using the False Discovery Rate (FDR) method to account for multiple comparisons associated with evaluating a large number of GO terms and to identify over-represented GO terms. GO terms that meet both the initial p-value cutoff of less than 0.05 and an FDR-adjusted p-value cutoff of less than 0.05 are considered significantly enriched. Since the specific functions of majority of date palm genes have not been explored, the current study utilized a functional inference approach to leverages orthology with the extensively studied Arabidopsis to predict the functions of *PdWRKYs*. The current study employed BLAST to compare the protein sequences of *PdWRKYs* with those in the NCBI database to identify their orthologs in *Arabidopsis* and establish a foundation for functional inference based on orthology.

### Plant material RNA extraction and RNA-seq data analysis

Healthy seedlings of the Khalas cultivar of date palm (*P. dactylifera*) were cultivated for three months under greenhouse conditions. During this period, plants were irrigated every second day under full light conditions to facilitate efficient water absorption by the plants. Experiments were conducted in growth chambers with a light cycle of 12 hr at 40 °C (08:00 -19:00) followed by 12 hr of darkness at 35 °C (20:00 -07:00), maintaining a relative humidity of 60%. The experiments were performed in two batches, following procedures described previously [63], using growth chambers manufactured by Excelsior Scientific, UK. Plants were irrigated every second day (“well-watered” in heat and control conditions) in the first set of experiments. After a two-week acclimatization period in the growth chamber, the plants were exposed to different growth temperatures (25°C for controlled conditions and 40 °C for high heat). Harvesting was conducted 6 hr after the onset of the first light. For the second batch, irrigation was discontinued after a two-week acclimatization period. Plants grown at 40 °C were harvested 5 days after irrigation was stopped, representing a combination of drought and heat stress. In contrast, the plants grown at 25 °C were harvested 8 days later under drought stress conditions. Due to the reduced relative humidity in the 40 °C chamber, water deprivation was maintained for a duration of 3 days for plants growing at 25 °C. For each experimental condition, four plants (biological replicates) were harvested for total RNA extraction. RNA extraction buffer was prepared according to the protocol described by [64], with the following composition: 0.25 M NaCl, 0.05 M Tris-HCl, 20 mM EDTA, 1% (w/v) sodium dodecyl sulfate (SDS), and 4% (w/v) polyvinylpyrrolidone (PVP). The extracted RNA was quantified using the Qubit 3.0 and the dsRNA broad range kit (Thermo Fisher, USA), and its quality was validated through 1% formaldehyde agarose gel electrophoresis. In our RNA-seq data analysis, we used previously submitted data to the NCBI Sequence Read Archive (SRA: ERX2220039-ERX2220054) under the same experimental conditions [63]. Raw reads were retrieved from the NCBI SRA repository, and their quality was assessed using FastQC software (https://www.bioinformatics.babraham.ac.uk/projects/fastqc/). A streamlined computational pipeline was implemented to identify gene regulation differences among control, drought and heat stressed plants. This pipeline included the following sequential steps: (1) FastQC https://www.bioinformatics.babraham.ac.uk/projects/fastqc, accessed on 4 March 2024) was used for quality checking of the sequencing data; (2) Trim Galore [65] was used for trimming the sequencing data to remove low-quality reads; (3) HISAT2 [66] was used for indexing the reference genome and aligning the trimmed reads to it; (4) Feature Count (subread_v2.0.2) was employed to quantify the number of reads mapped to each gene; and (5) differential gene expression analysis was performed in the R program utilizing DESeq [67].

### Gene expression analysis by quantitative real time PCR (qRT-PCR)

A High-Capacity cDNA Reverse Transcription kit (Thermo-Fisher Scientific) was used to synthesize cDNA from 1 µg of the extracted RNA. The Polymerase Chain Reaction (PCR) was set with the following specific temperature settings: an initial denaturation at 25°C for 10 min, reverse transcription at 37°C for 2 hr, and inactivation at 85°C for 5 min. After PCR completion, the synthesized cDNA was stored at -20°C refrigerator for future use in qRT-PCR analysis. The expression levels of seven genes LOC103713231 (*WRKY28*), LOC103721327 (*WRKY72*), LOC103721580 (*WRKY50*), LOC120110335 (*WRKY70*), LOC103723396 (*WRKY35*), LOC103718774 (*WRKY1*), and LOC103707788 (*WRKY71*) were evaluated in triplicate using quantitative real-time PCR (qRT-PCR) with Applied Biosystems Life Technologies' Quant-Studio 5.0 system. These genes were selected based on their differential expression in our RNA-seq data analysis, which revealed their involvement in heat and drought stress tolerance. To evaluate the expression level of the selected genes specifically designed primers (Table S2) and the "SYBR" green Master Mix was used. The actin gene was used as a reference for all primers, and a threshold of 0.1 was established for gene amplifications. The qRT-PCR reaction was performed with the following parameters: Stage 1 included an initial denaturation at 94°C for 10 min, followed by 35 cycles of amplification at 94°C for 45 s, 65°C for 45 s and 72°C for 1 min and the final extension step at 72°C for 10 min. PCR reactions were conducted in triplicate to ensure accuracy. A threshold level of 0.1 was set for the analysis of gene amplifications.

## Results

### Genome-wide identification of the PdWRKY genes

This study has curated 73 *PdWRKY*, 101 *OsWRKY*, 72 *AtWRKY* and 131 *EgWRKY* non-redundant TFs obtained from different databases including PlantTFDB v.5.0, iTAK and NCBI. The iTAK IDs were substituted with LOC accession numbers through the comparison of their respective downloaded sequence using a BLAST search in NCBI (Table S1). The LOC accession numbers of the *PdWRKY* TFs were used as queries to obtain the corresponding genome and protein sequences from the NCBI. The alternate splice variants of these TFs were neglected due to a lack of understanding or availability of their specialized regulatory roles [68].

### Alignment of multiple sequences and phylogenetic tree analysis

**Fig. 1:**
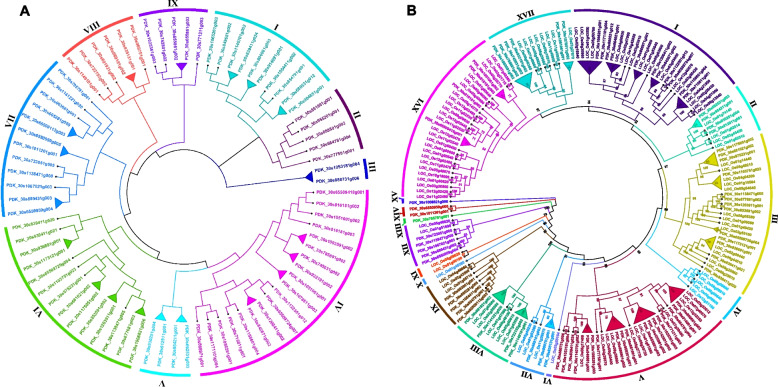
**A** The phylogenetic tree of 73 *P. dactylifera* WRKY TFs was constructed by ML method using MEGA-11 software with 1,000 bootstrap replicates. The major nine phylogenetic groups are marked as I to IX respectively. **B** Joined phylogenetic tree constructed from an alignment of 73 *P. dactylifera* (*PdWRKYs*) and 101 *O. sativa* (*OsWRKYs*) protein sequences by the ML method with bootstrapping (1,000 replicates) using the MEGA 11 software. The resulting 17 groups are shown in different colors

**Fig. 2:**
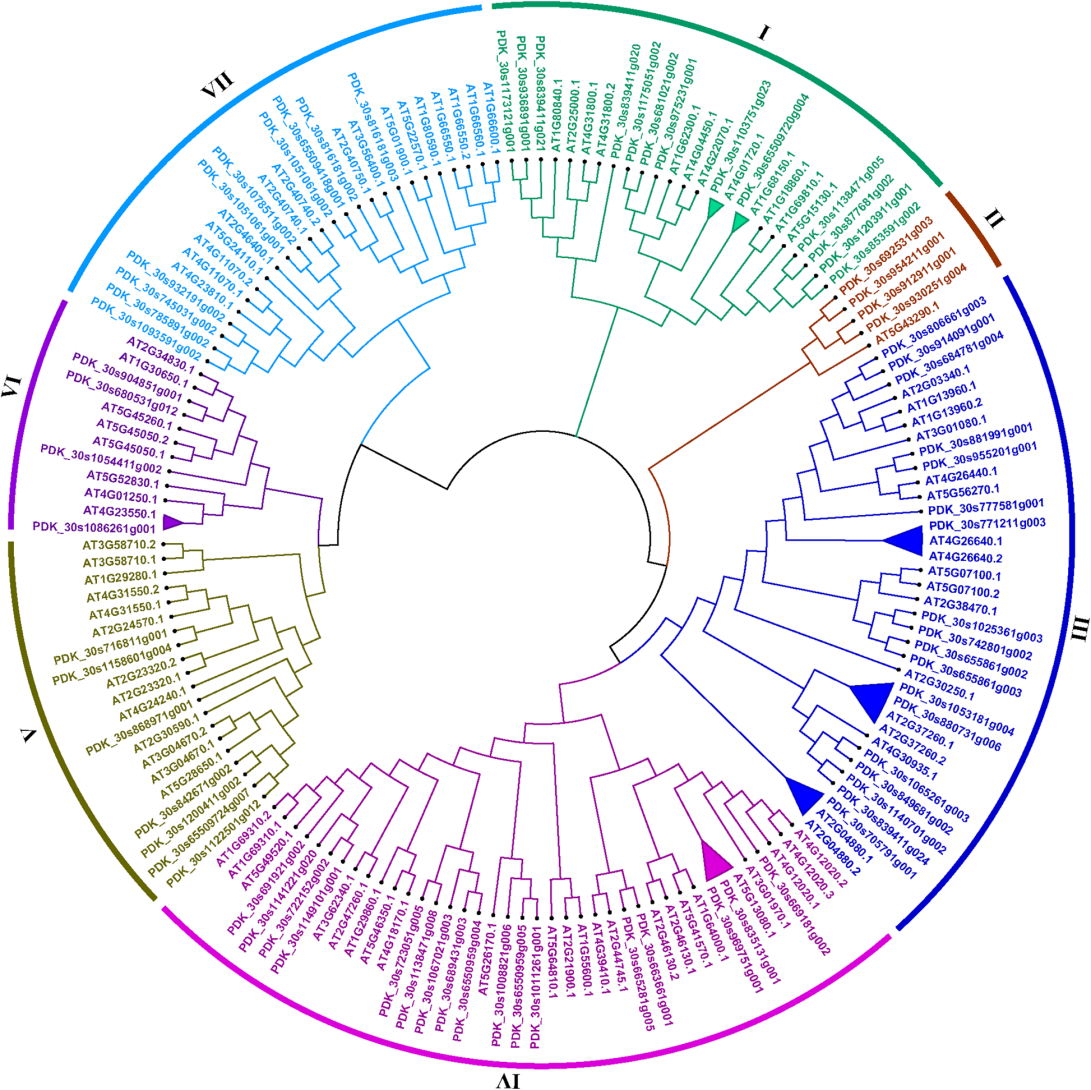
Joined phylogenetic tree constructed from an alignment of 73 *P. dactylifera* (*PdWRKYs*) and 72 *A. thaliana* (*AtWRKYs*) protein sequences by the ML method with bootstrapping (1,000 replicates) using the MEGA 11 software. The resulting 7 groups are shown in different colors

Figure 1A depicted 9 major clades (clade I- clade IX) based on sequence similarity and phylogenetic tree topology of the *PdWRKY* TFs. The findings revealed that clade-IV had the maximum *PdWRKY* members (19), followed by clade-VI with 14 members while clades-III, V, VIII, and IX had 2, 4, 6, and 5 *WRKY* TF members respectively. Minimum (2) number of *PdWRKYs* were found in Clade-III. To better comprehend the evolutionary relationships among the *WRKY* TFs of *P. dactylifera* and *O. sativa*, we also constructed a phylogenetic tree combining *PdWRKYs* and *OsWRKYs*. The construction of a topological tree and subsequent classification of *P. dactylifera* and *O. sativa WRKY* TFs revealed 17 distinct phylogenetic clades. Among these, clade-I, V and XVI are the largest ones, each with 29 WRKY members, followed by clade-III, with 26 members. Clades-VI, X, XIII, and XV, on the other hand, have only one member each, whereas clades-XI and XIV have two members of the *WRKY* TFs (Fig. 1B). The phylogenetic tree combining *PdWRKY* and *AtWRKY* sequences is divided into 7 major clades. The largest among these is clade-IV, encompassing 40 members, while the smallest one is clade-II, which contain 5 members (Fig. 2). Moreover, most of the paralogous pairs are found in the clade-III while there are no orthologous pairs in clade II, V and VII implies. The absence of the orthologous pairs in these clades indicates that the *WRKY* genes in *P. dactylifera* and *A. thaliana* have evolved independently and may have different functions. To strengthen our homological analysis, we also constructed a combined phylogenetic tree from *PdWRKYs*, *OsWRKYs* and *EgWRKYs*. In this tree *PdWRKY* form 45 orthologous pairs with *EgWRKYs* indicate their close evolutionary and potentially functional relationship. On the other hand, the lower number (only 2) of orthologous pairs of *PdWRKYs* with *OsWRKYs* indicates greater evolutionary divergence between them (Figure S1).

### Insights from the paralogous and orthologous relationships

Paralogous and orthologous genes represent two different types of homologous genes. Paralogous genes originate from gene duplication events within the same genome, while orthologous genes exist in different species and have evolved from a common ancestral gene through the process of speciation, retaining similar functions inherited from their shared ancestor [69, 70]. Previous studies have demonstrated that paralogous proteins often retain their biological functions, even when their target regions change. In contrast, orthologs generally continue to perform similar roles in different organisms [71]. Therefore, conducting comprehensive comparative genome-wide analyses of paralogous and orthologous gene pairs is one of the most effective techniques used to identify the functional roles of uncharacterized genes [72].

Putative paralogous pairings of *PdWRKY* TFs were identified by analyzing the tree depicted in Fig. 1A. A detailed analysis of the constructed trees revealed 19 paralogous pairs of *PdWRKY* proteins, supported by strong bootstrap (BS) scores (>70), as indicated by the triangular cartoons. A comparative phylogenetic analysis of *WRKY* proteins in *P. dactylifera* and *O. sativa* revealed that approximately 52% of *PdWRKYs* have orthologous pairing with *OsWRKYs*. These orthologous pairings are supported by a strong degree of homology indicating a high level of sequence similarity and evolutionary relatedness between the *WRKY* proteins in the two species (Fig. 1B). In the combined phylogenetic tree of *P. dactylifera* and *Arabidopsis* only 9 *PdWRKYs* form orthologous relationship with *AtWRKYs*, suggests that the majority of *WRKY* genes in these two species evolved differently, possibly resulting in unique functions or regulatory roles.

### Gene function prediction based on homology

Genes with similar functional roles are thought to have evolved from a common ancestral gene. Therefore, homology analysis is commonly used to predict the functions of uncharacterized genes by comparing them with known genes with annotated functions [73]. Since specific physiological and regulatory roles of most *WRKY* TFs in *P. dactylifera* have not been extensively investigated, a comparative homology-based analysis of these genes with their well-studied counterparts in *O. sativa* and *A. thaliana* will help us in predicting their specific functional roles. For instance, in *O. sativa* LOC_Os02g16540 (*OsWRKY39*), LOC_Os06g30860 (*OsWRKY31*) the orthologs of PDK_30s1086261g001 (*WRKY22*) and PDK_30s1171101g004 (*WRKY22*) induced expression of drought and cold stress related genes, respectively [74, 75]. Combinedly constructed phylogenetic tree of *P. dactylifera* and *A. thaliana* showed that only 9 *PdWRKYs* form orthologous relationships with *AtWRKYs*. The PDK_30s1103751g023 form strong an orthologous pair with AT4G01720.1 (*AtWRKY47*) gene in Arabidopsis which has been investigated for its crucial involvement in regulating plant tolerance to boron toxicity [76]. The PDK_30s771211g003 make an orthologous relationship with AT4G26640.2 (*AtWRKY20*), a transcriptional activator involved in sucrose-induced starch biosynthesis [77]. In clade-IV, PDK_30s835131g001 and PDK_30s969751g001 (*WRKY47*) form strong orthologous relationship with AT5G13080.1 (*AtWRKY75*), a key transcriptional regulator involved in the defense signaling pathways triggered by salicylic acid (SA) and jasmonic acid/ethylene (JA/ET). This regulation is crucial for Arabidopsis in defending against *Sclerotinia sclerotiorum* and coping with oxalic acid stress [78] (Fig. 2). Hence, by leveraging these homology relations, the physiological functions of their uncharacterized counterparts can be deduced. This comparative approach enabled us to extrapolate functional insights from the well-studied genes to related counterparts that have not yet been extensively characterized.

### Analysis of exon-intron architecture and conserved motifs

**Fig. 3:**
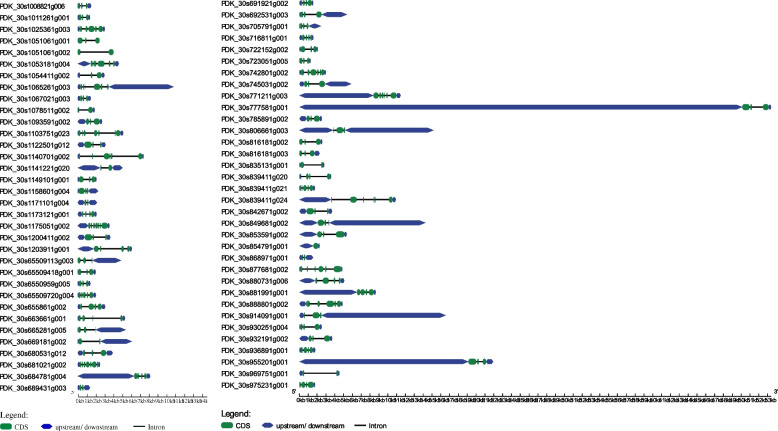
Exon-intron structure analysis of *P. dactylifera WRKY* genes was performed by GSDS database. The blue boxes indicate upstream/downstream, the green boxes indicate exons, and the black lines indicate introns

**Fig. 4:**
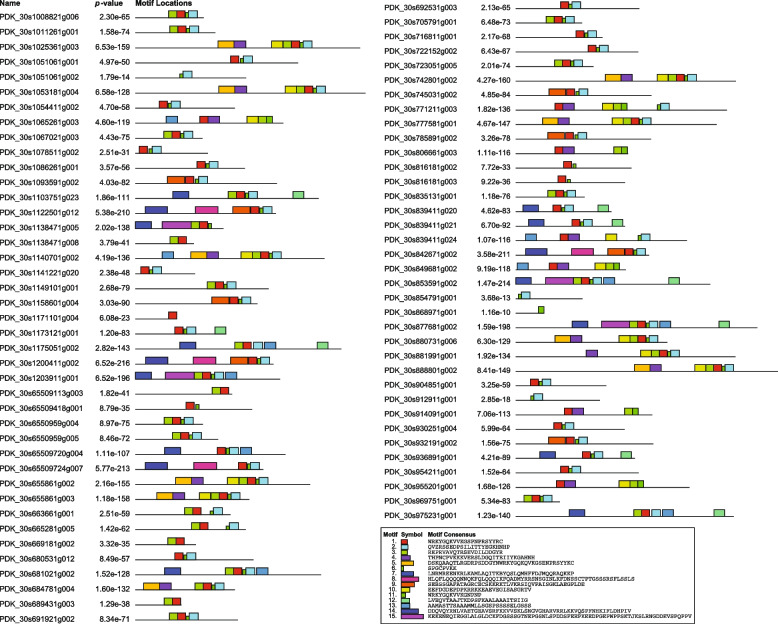
The most conserved common motifs of *PdWRKY* TFs family were identified by MEME database with the complete amino acids sequences. The light blue colored motif signifies the WRKY motif

Analyzing gene structure and composition can provide valuable supporting evidence for understanding evolutionary patterns and gene duplication events [79]. The exon-intron organization maps for *PdWRKY* genes were generated using the Gene Strcuture Display online tool (Fig. 3). The results revealed that the majority (86.7%) of *PdWRKY* genes have more than 2 and relatively long introns. This evidence suggests that *PdWRKY* genes have remained relatively conserved in terms of their gene structure and organization over a significant evolutionary timescale as newly evolved species tend to have fewer and short introns compared to their ancestors [80–82]. To investigate the compositional diversity among *PdWRKY* gene members, MEME software was used to identify 15 different conserved motifs within the proteins (Fig. 4). The light, blue-colored motif was consistently detected in virtually all of the *PdWRKY* proteins, indicating that it represents the conserved *WRKY* domain. The potentially conserved motif, indicated by light blue coloring, was not identified in approximately 13% of the members, including PDK_30s1171101g004 (*WRKY22*), PDK_30s65509113g003 (*WRKY13*), PDK_30s65509418g001(*WRKY70*), PDK_30s669181g002 (*WRKY75*) PDK_30s689431g003(*WRKY71*), PDK_30s806661g003 (*WRKY4*), PDK_30s816181g002 (*WRKY70*), PDK_30s816181g003 (*WRKY70*), PDK_30s849681g002 (*WRKY4*) and PDK_30s914091g001(*WRKY4*).

### Analysis of chromosomal distribution and gene duplication

**Fig. 5:**
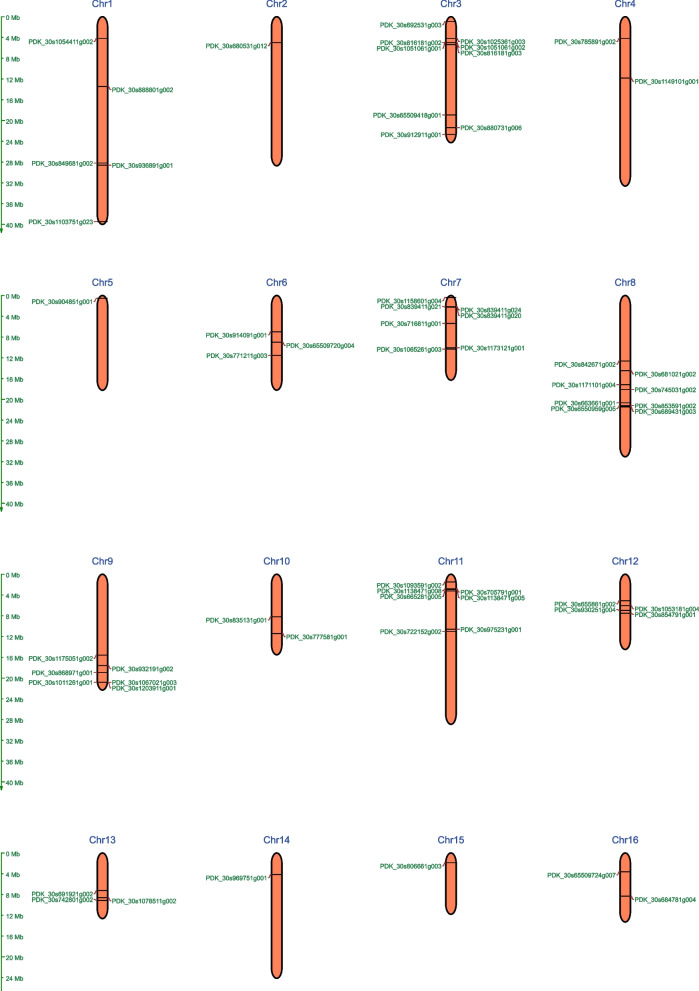
Chromosomal localizations of *PdWRKY* genes, identified on 16 out of the 36 chromosomes of *P. dactylifera*

**Fig. 6:**
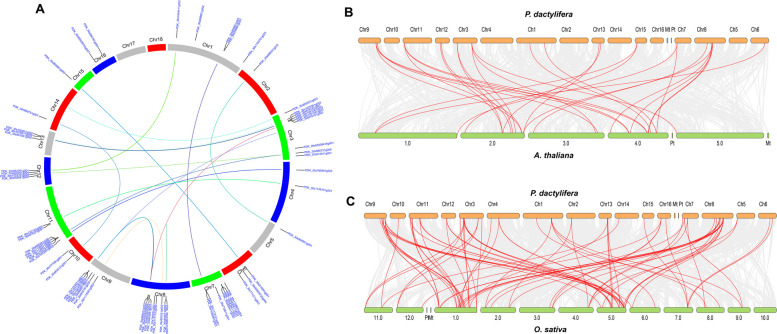
Synteny analysis and duplication of *PdWRKY* genes. **A** Chromosomal positions and interchromosomal groups of duplicated *PdWRKY* gene pairs across the 18 chromosomes (Chr1–Chr18). Lines represent duplicated gene pairs. **B** Synteny analysis between *PdWRKY* and *AtWRKY* genes. (C) Synteny between *PdWRKY* and *OsWRKY* genes. Note: Chromosome names in *P. dactylifera* were renamed to Chr1, Chr2, etc., as shown in Table S4

According to the chromosomal localization analysis, the identified *PdWRKY* genes are exclusively distributed only on 16 chromosomes of *P. dactylifera*. The online tool MG2C was used to map the genes to their respective chromosomal locations. As shown in Fig. 5, chromosome 3 contained the maximum number (14.75%) of *PdWRKY* genes, followed by chromosome 8 with 13.11% *PdWRKY* genes. Figure 6A illustrates the chromosomal distribution and interchromosomal duplication of the *PdWRKY* gene family across the 18 chromosomes of *P. dactylifera*. A total of 73 *PdWRKY* genes were identified and mapped across the chromosomes. These genes are primarily distributed across 16 chromosomes, with notable concentrations on chromosomes 3, 8, and 9. Chromosome 3, in particular, contains the largest number of *PdWRKY* genes, suggesting significant evolutionary dynamics on this chromosome. Tandem duplications, which are indicative of segmental duplications, were predominantly found on chromosomes 3 and 9. Notably, the following gene pairs were identified as tandem duplicates: PDK_30s816181g002 (*WRKY70*) and PDK_30s1051061g001 (*WRKY55*) on chromosome 3, and PDK_30s1067021g003 (*WRKY51*) and PDK_30s1203911g001 (*WRKY72*) on chromosome 9.

The synteny analysis identified 18 *PdWRKY* genes that show conserved syntenic relationships with *AtWRKY* genes (Fig. 6B). These collinear blocks are represented by gray lines, and the orthologous gene pairs are highlighted in red. The identification of these conserved orthologous relationships indicates that these *PdWRKY* genes likely perform similar roles in regulating stress responses, despite the evolutionary divergence between monocots and dicots. Similarly, Figure 6C displays the synteny analysis of the *PdWRKY* genes in relation to *O. sativa*. This comparison reveals a higher number of red lines, reflecting a broader syntenic relationship between the two species. The increased number of red lines in this panel suggests that multiple *PdWRKY* genes have conserved synteny with more than one *OsWRKY* gene. This observation likely arises from the genomic complexity of rice, which exhibits a higher degree of gene duplications and more extensive collinearity within its genome.

### Analysis of cis-regulatory elements

The upstream region of the *WRKY* genes in *P. dactylifera* harbors a variety of cis-regulatory elements linked to diverse biological functions. A total of 13 consensus cis-elements were identified within the 2000 bp upstream region, each associated with distinct regulatory roles (Figure S2). Among these, several elements are involved in hormonal regulation during stress, including AuxRR and TGA (auxin responsiveness), ABRE (abscisic acid responsiveness), MeJA (jasmonic acid responsiveness), TCA (salicylic acid responsiveness), as well as GARE and TATC (gibberellin responsiveness). Additionally, GT1 and G-Box elements were identified as being responsive to light, while the MBS element was associated with drought stress. Other elements, such as DRE (responsive to dehydration, low temperature, and salt stress), LTR (low-temperature stress), and TC (defense and stress), were also found, highlighting the gene's involvement in abiotic stress responses. Notably, specific *WRKY* genes, such as LOC103702844 (*WRKY44*) and LOC103704540 (*WRKY1*), contain a higher number of motifs related to the abiotic stress hormones ABA and JA, respectively. Furthermore, several *WRKY* genes, including LOC103698294 (*WRKY35*), LOC103698690 (*WRKY4*), LOC103698763 (*WRKY75*), LOC103706248 (*WRKY70*), LOC103705538 (*WRKY72*), LOC103705233 (*WRKY4*), and LOC103704540 (*WRKY1*), were found to possess motifs associated with drought stress. Only one gene, LOC103708338 (*WRKY53*), contained a motif linked to dehydration and osmotic stress. The diversity of cis-elements identified in the *WRKY* gene family of *P. dactylifera* underscores their responsiveness to a wide range of environmental stimuli (Table S3).

### Functional annotations based on protein-protein interaction networks

**Fig. 7:**
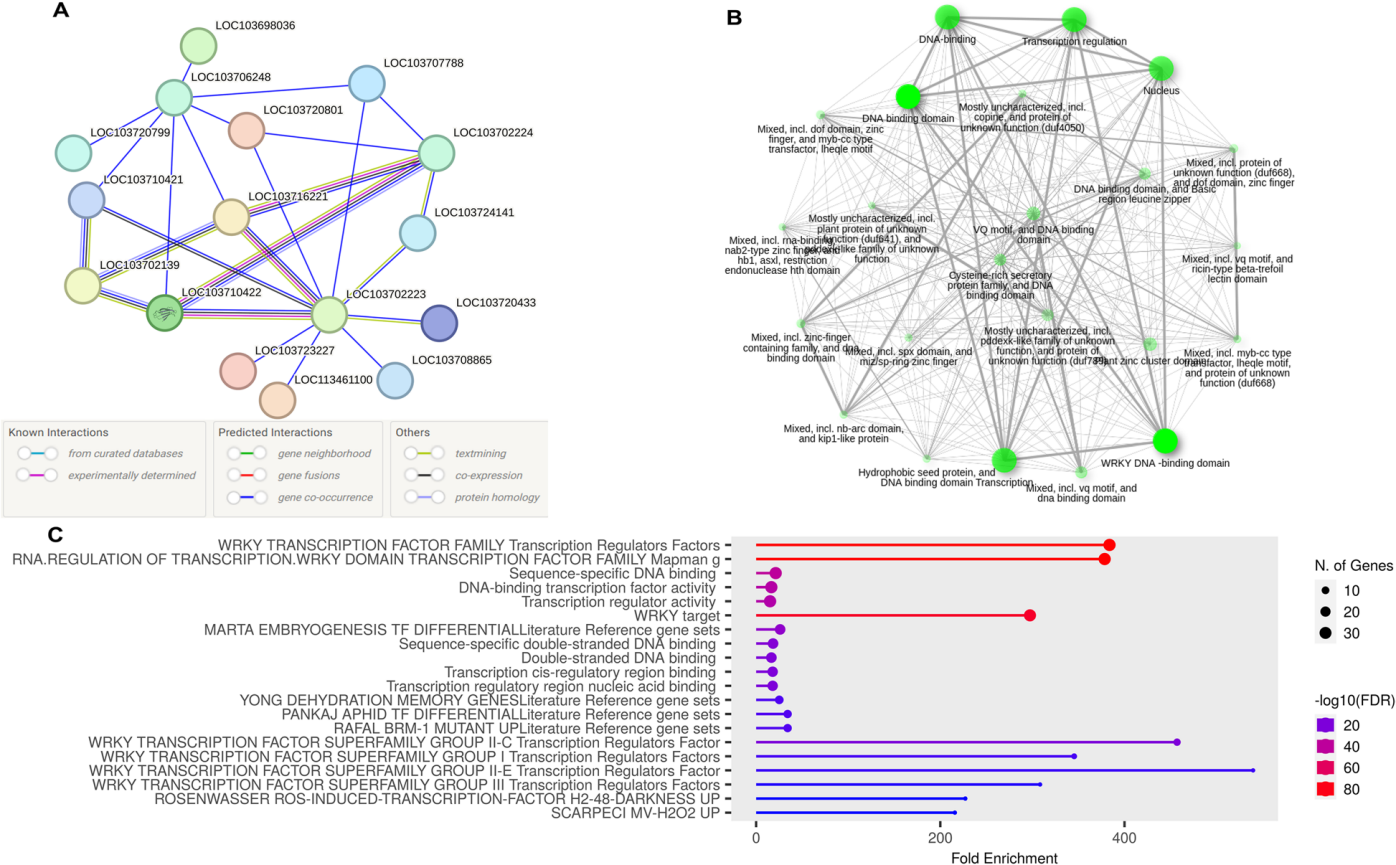
**A** Protein-protein association network of the *PdWRKY* genes based on their available information. The online tool STRING was used to predict the entire network. Different line colors represent the type of evidence for the associations, which are shown in legend. **B** Gene ontology (GO) enrichment of the *PdWRKY* genes. The size of the circles represents the number of genes in each category. **C** GO and KEGG enrichment analyses of *PdWRKY* genes and their expression patterns

Protein-protein interactions are essential for regulating gene expression in response to specific environmental conditions. Proteins that frequently interact with each other are more likely to participate in similar biological processes, suggesting that they have similar functional annotations [60, 83]. The protein-protein interaction networks of differentially expressed genes were analyzed using the online STRING database. The results revealed that LOC103702224 (PDK_30s1051061g002/*WRKY70*), LOC103710421 (PDK_30s839411g020/*WRKY40*), LOC103716221(PDK_30s936891g001/*WRKY71*) LOC103702223 (PDK_30s816181g002/*WRKY70*) and LOC103710422 (PDK_30s839411g021/ *WRKY76*) are the hub proteins in the whole networks. Most of the *PdWRKY* proteins showed sequence homology, gene fusions and co-occurrence; however, only a few proteins are linked with each other directly. The majority of these interactions were further validated by the constructed phylogenetic trees. For instance, LOC103702224 (PDK_30s1051061g002/*WRKY70*) LOC103710421 (PDK_30s839411g020/ *WRKY40*), LOC103710422 (PDK_30s839411g021/*WRKY76*) and LOC103716221 (PDK_30s936891g001/ *WRKY71*) belong to clade-III of the phylogenetic tree. Thus, this approach can be used to predict the potential functional roles and pathways in which the uncharacterized genes might participate by associating them with their already studied counterparts. For instance, LOC103702224 (*WRKY70*) is ortholog of LOC_Os05g25770 (*OsWRKY45*) which plays a crucial role in defense mediated by salicylic acid during various stress conditions in rice [84]. Additionally, LOC103702224 (*WRKY70*) has a strong co-occurrence and gene fusion links with un-characterized *PdWRKY* members including LOC103716221(*WRKY71*), LOC103710422(*WRKY76*) and LOC103702223 (*WRKY70*) so we can hypothesize potential functional roles for these TFs based on the interactions and associations with LOC103702224 (*WRKY70*) (Fig. 7A).

### Gene ontology analysis

To further understand the functional roles of *PdWRKY* genes, GO enrichment analysis was performed using the online GO tool. The findings revealed that most of the genes were predominantly associated with molecular functions (MF) and cellular components (CC), such as DNA binding (GO: 0003677), transcription (GO: GO:0006350), regulation of DNA-templated transcription (GO: 0006355) and nuclear component (GO:0005634) (Fig. 7B and 7C). To enhance the depth of our analysis, we extended our investigation to include a GO enrichment analysis of orthologous pairing of *PdWRKYs* with *AtWRKYs* (Figure S3). Among the 54 corresponding annotated orthologous of *PdWRKYs* and *AtWRKYs*, we observed that 37 (50.68%), 12 (16.43%), and 5 (6.84%) of the genes were assigned to BP, MF, and CC, respectively. GO annotation results for orthologs of *PdWRKYs* in *AtWRKYs* revealed several significantly enriched terms. Highly enriched terms in the BP category include regulation of RNA biosynthesis process (GO:2001141), regulation of DNA template transcription (GO:0006355), defense response against bacteria (GO:0042742), and jasmonic acid-mediated signaling pathway (GO:0009867), response to salicylic acid (GO:0009751), and leaf senescence (GO:0010150). The most abundant terms within the MF class included sequence-specific DNA binding (GO:0043565), transcriptional regulatory activity (GO:0140110), and DNA-binding transcription factor activity (GO:0003700). Intracellular organelles (GO:0043229) and intracellular anatomical structures (GO;0005622) were two of the most enriched terms in the CC class. Furthermore, KEGG pathway enrichment analysis indicated considerable enrichment in pathways such as plant-pathogen interaction (KEGG:04626) and MAPK signaling (KEGG:04016). In brief, the GO and KEGG pathway enrichment analyses validate the *PdWRKY* genes' functional relevance in various biological, molecular, and cellular processes, including gene expression regulation, interactions with plant pathogens, stress responses, and the biosynthesis of various important metabolites.

### Differential expression analysis of PdWRKY genes under drought and heat stress

**Fig. 8:**
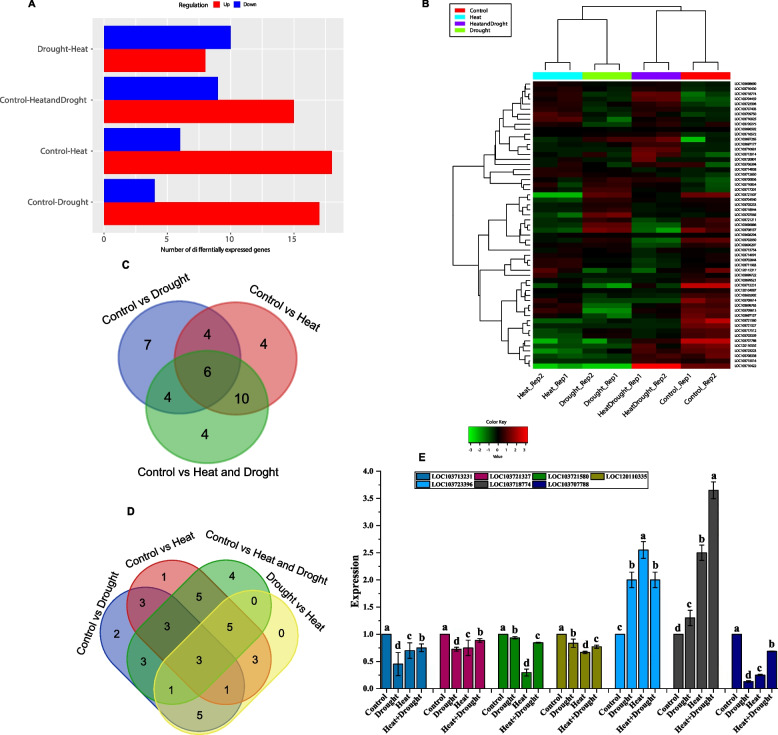
**A** Statistical analysis of differentially expressed genes (DEGs) under individual heat and drought stress, as well as under the combined stress of heat and drought in *P. dactylifera*. **B** Heatmap of differentially expressed *PdWRKY* genes under individual heat and drought stress, as well as under the combined stress of heat and drought in *P. dactylifera*. **C, D** Venn diagrams of the differentially expressed *PdWRKY* genes under individual heat and drought stress, as well as under the combined stress of heat and drought in *P. dactylifera*, **E** qRT-PCR analysis of DEGs genes under individual heat and drought stress, and combined stress conditions in *P. dactylifera*

RNA-seq data utilization is a powerful approach to study genes expression patterns and their specific physiological functions during stress conditions that can advance our understanding of stress tolerance mechanisms in different crop species [85]. Therefore, in the current study, we evaluated the expression levels of the *PdWRKY* genes and their functional roles in *P. dactylifera* under drought and heat stress conditions using RNA-seq data. Our results revealed that more than 7 genes (12.2%) and 10 (17.5%) of the investigated *PdWRKY* genes exhibited significant up and down regulation, respectively in response to heat stress compared to drought stress. As depicted in the Fig. 8A and 8B in the combined stress condition of heat and drought, approximately 15 genes (26.3%) exhibited upregulation, while about 9 genes (15.78%) of the investigated genes showed downregulation when compared to the expression levels of *WRKY* genes in the control condition of *P. dactylifera*. For instance, the genes LOC103718774 (PDK_30s65509724g007/ *WRKY1*), LOC103704459 (PDK_30s1141221g020/*WRKY57*), LOC103697265 (PDK_30s692531g003/*WRKY49*), LOC103710681 (PDK_30s888801g002/*WRKY2*), LOC103708338 (PDK_30s1093591g002/*WRKY53*) and LOC103710422 (PDK_30s839411g021/*WRKY76*) showed upregulation while LOC103696886 (PDK_30s691921g002/*WRKY48*), LOC103708157 (PDK_30s932191g002/*WRKY41*), LOC103702950 (PDK_30s745031g002/*WRKY41*) and LOC103709614 (PDK_30s1011261g001/*WRKY51*) were downregulated.

In individual heat stress conditions, the highest number of *PdWRKY* genes exhibited upregulation compared to other conditions i.e., about 18 (31.5%), while only 6 (10.5%) of the genes showed downregulation. For instance, LOC103709750 (PDK_30s663661g001/*WRKY13*), LOC103710925 (PDK_30s1171101g004/*WRKY22*), LOC120112317 (PDK_30s705791g001/*WRKY51*) and LOC103709613 (PDK_30s1067021g003/*WRKY51*) significantly showed upregulation while LOC103721507 (PDK_30s742801g002/ *WRKY24*), LOC103707065 (PDK_30s716811g001/*WRKY51*), LOC103707788 (PDK_30s1173121g001/*WRKY71*), LOC103723223 (PDK_30s723051g005/ *WRKY50*) and LOC103710422 (PDK_30s839411g021/*WRKY76*) were found to be downregulated. Similarly, in individual drought stress more than 15 (26.3%) *PdWRKY* showed upregulation while approximately 4 (7%) genes exhibited downregulation as compared to control conditions. During drought stress, the expression patterns of most genes tend to contrast with those observed during the heat stress. For instance, LOC103721507 (PDK_30s742801g002/*WRKY24*), LOC103707065 (PDK_30s716811g001/*WRKY51*), LOC103708157 (PDK_30s932191g002/ *WRKY41*) exhibited upregulation, whereas LOC103710925 (PDK_30s1171101g004/*WRKY22*), LOC120112317 (PDK_30s705791g001/*WRKY51*), LOC103709613 (PDK_30s1067021g003/*WRKY51*) and LOC103710422 (PDK_30s839411g021/*WRKY76*) showed downregulation. The expression patterns of most of the *PdWRKY* genes in stress conditions change significantly in response to heat and drought stress compared to the control condition. This observation indicates that these *PdWRKY* genes likely play a key role in the plant’s response to these environmental stressors. To identify the genes that may function together in different stress responsive pathways, the *PdWRKY* genes were divided into four clusters (Figure S4). Cluster-A, with its higher number of genes (30 in total), may represent a diverse set of *PdWRKY* genes that are involved in various aspects of the stress response pathways. The larger size of this cluster suggests that it may encompass genes with both overlapping and different functions in response to drought and heat stress. On the other hand, Cluster-D, with only one *PdWRKY* gene, indicates that this particular gene has a unique role in stress responses compared to the genes in other clusters. Overall, 39 *PdWRKY* genes were differentially expressed in both drought and heat stress. In individual drought and heat stress conditions, 21 and 24 genes were dietetically expressed, respectively. However, only 16 genes exhibited differential expressions across all stress conditions (drought, heat and combined drought and heat). Plant responses to stressors are expected to be coordinated by the 6 genes that exhibited significant differential expression under all stress conditions (Fig. 8C and 8D).

### Validation of genes expression by qRT-PCR analysis

To validate the accuracy of the RNA-seq data, we used specifically designed primers to perform qRT-PCR analysis of selected seven LOC103713231 (*WRKY28*), LOC103721327 (*WRKY72*), LOC103721580 (*WRKY50*), LOC120110335 (*WRKY70*), LOC103723396 (*WRKY35*), LOC103718774 (*WRKY1*), and LOC103707788 (*WRKY71*) (Table S2). The qRT-PCR expression profiles matched those observed in the RNA-seq data, indicating a significant relationship between them. This consistency demonstrates the reliability of our RNA-seq data (Fig. 8E).

## Discussion

In the current study a total of 73 *PdWRKY* genes were identified in the *P. dactylifera* genome thorough in various *in-silico* genome-wide analyses. Although the functional roles of most of these genes have not yet been defined, our findings offer insight into their potential significance in drought stress. To gain a better understanding of the regulatory mechanisms underlying the response to heat stress and drought, we conducted a comprehensive investigation of the identified genes. This investigation included an analysis of phylogeny, gene structure, conserved motifs, chromosomal localization, gene ontology, protein-protein interactions, and differential gene expressions under drought and heat stress conditions. These investigations provided us with vital insights into the *PdWRKY* family's functional repertoire, with *O. sativa* and *A. thaliana* serving as comparison plants. To assess evolutionary and functional insights, phylogenetic trees were constructed that combined *P. dactylifera WRKY* TFs with their counterparts in *O. sativa* and *A. thaliana*. The greater the sequence similarities, the more likely the proteins are to perform similar functional roles in the plant species [86, 87]. The comparative phylogenetic tree analysis of *OsWRKYs* and *PdWRKYs* revealed 17 main clades differentiated by tree topologies and sequence similarities. Within these clades, we found that *PdWRKY* genes were present in 14 clades but absent in the remaining 3 clades of the constructed trees. The analysis indicates that 19 pairs of *PdWRKY* genes were identified to have a paralogous relationship supported by high BS scores. Additionally, it has been demonstrated that approximately 52% of *PdWRKY* genes have orthologous pairs with *OsWRKY* genes, suggesting ancestral relationships between *P. dactylifera* and *O. sativa WRKY* genes before the divergence of the species, as previously reported [88]. LOC_Os01g14440 (*OsWRKY1*), making orthologous pair with uncharacterized PDK_30s975231g001 (*WRKY31*), PDK_30s681021g002 (*WRKY31*) and PDK_30s1175051g002 (*WRKY31*), is associated in host defense activation against biotic stress induced by red seaweed in rice plants [89]. The uncharacterized *P. dactylifera* gene, PDK_30s1103751g023 (*WRKY47*) form orthologous pairing with three *OsWRKY* TFs (LOC_Os04g04300, LOC_Os01g18584 and LOC_Os05g04640) which are known to be involved in the regulation of adaptation to abiotic stresses, especially drought stress [90–92]. LOC_Os11g29870 (*OsWRKY72*), an ortholog of PDK_30s969751g001 and PDK_30s835131g001, could serve as a convergence point for auxin transport and ABA signaling pathways. This convergence most likely plays an important role in balancing developmental processes and defense systems, contributing to salinity tolerance [93, 94]. LOC_Os05g25770 (*OsWRKY45*) ortholog of PDK_30s1051061g002 and PDK_30s816181g003 plays a crucial role in defense mediated by salicylic acid (SA) during salt, drought, and cold stresses in rice [84, 95]. Similarly, in the combined phylogenetic tree of *PdWRKYs* and *AtWRKYs* only nine orthologous pairs were identified, indicating that the majority of *WRKY* genes in these two species have followed distinct evolutionary paths. Conserved protein motifs and gene structure analyses were employed to validate the accuracy of the phylogenetic classification. According to the conserved motifs study, all members of *PdWRKY*, contain the same domain (QVZRSSEDPSILITTYEGKHNHP), with a few exceptions, and the composition of motifs in each subfamily was found to be similar. These exceptions may result from various factors, such as a lack of homology, genomic rearrangements, or interruptions in the alignment process [96]. In contrast, several *WRKY* proteins exhibited only one or two motifs, possibly due to their short lifespan, which may have resulted in the truncation of their domains. With a few exceptions, most genes within the same phylogenetic group have similar exon-intron structures and motif compositions, with the number and positions on introns being almost completely conserved within most of the subfamilies. For instance, approximately 73% of *PdWRKY* members in clade-I possessed three exons, with 82% of them containing three to six conserved motifs. Similarly, all members of clade-II in the phylogenetic tree contain three exons, with 75% of them containing three conserved motifs. A few *PdWRKY* proteins were found to have a unique structure, characterized by fewer motifs and the absence of the signature motif present in other members. It was observed that a few *PdWRKY* proteins are unique in structure with fewer motifs, and lack the characteristic motif found in other members. This diversity in *PdWRKY* proteins could imply functional differences, perhaps in the expression regulation of specific genes [97]. It was discovered that *PdWRKY* members have introns and possess complex structural organization which suggest that these have an important role to maintain variability in gene expression and splicing of conserved genes, potentially contributing to the adaptability and responsiveness of plants to their environment [98, 99]. Gene duplication events play a significant role in creating genetic novelty and acquiring new gene functions in organisms [100], with tandem duplications, indicative of segmental duplications, being predominantly observed on chromosomes 3 and 9. The synteny analysis identified conserved orthologous relationships between *PdWRKY* and *AtWRKY* genes, indicating that they may evolved from a common ancestor and share similar roles in stress regulation, as supported by previous study [101]. The promoter regions of *PdWRKYs* were found to contain 13 cis-elements with multiple potential regulatory functions. Elements like AuxRR and TGA, associated with auxin signaling, are consistent with [102] findings on the role of auxin in plant growth and stress adaptation. The ABRE element, which responds to ABA, is consistent with the research by [103] on the importance of ABA in abiotic stresses. Similarly, the MeJA and TCA elements, associated with JA and SA signaling, are consistent with previous studies demonstrating their roles in pathogen defense and stress management [104, 105].The MBS, DRE, and LTR elements, which are associated with drought, dehydration, and low temperature stress respectively, support the previous studies on the role of WRKY genes in managing abiotic stresses [106].The protein-protein interaction networks suggest that most of the *PdWRKY* proteins showed protein homology, fusions and co-occurrence which indicate potential functional relationships among them. This approach can be used to predict the potential roles of the uncharacterized genes by associating them with well-studied counterparts. For instance, LOC103702224 (*WRKY70*) is identified as an ortholog of LOC_Os05g25770 (*OsWRKY45*), which is known to play a crucial role in defense mediated by salicylic acid during stress conditions in rice [84]. Therefore, LOC103702224 (*WRKY70*) may have similar functions, and its strong interactions with other uncharacterized *PdWRKY* members (LOC103716221(*WRKY71*), LOC103710422 (*WRKY76*), and LOC103702223 (*WRKY70*) suggest similar potential functional roles for these TFs. The study revealed that chromosomes 3 and 8 have the highest percentage of *PdWRKY* genes with 14.75% and 13.11% respectively among the sixteen analyzed chromosomes. This information suggests that these two chromosomes may have a significant role in the date palm’s genetic makeup and might be crucial for the functional specialization within the date palm genome. The conducted GO enrichment analysis of *PdWRKY* genes provides valuable insights into the functional roles of these genes. The majority of the genes were found to be primarily associated with crucial molecular functions and cellular components, including DNA binding, transcription, regulation of DNA-templated transcription, and nuclear components. This aligns with existing literature on *WRKY* genes, which highlights their involvement in transcriptional regulation and DNA binding activities [107]. Expanding the analysis to include the orthologous pairing of *PdWRKYs* with *AtWRKYs* further enriches our understanding. Identification of highly enriched terms related to the regulation of RNA biosynthesis, defense responses to bacteria, and signaling pathways associated with jasmonic acid and salicylic acid highlight the diverse functions of *WRKY* genes in various biological contexts [108]. The KEGG pathway enrichment study complements the GO analysis by highlighting specific pathways associated with *PdWRKY* genes. The significant enrichment of pathways related to plant-pathogen interaction and MAPK signaling supports the involvement of *PdWRKY* genes in plant defense mechanisms and stress responses [109]. These findings are consistent with the well-defined functional roles of *WRKY* genes in regulating plant responses to various biotic and abiotic stresses [110, 111]. Transcriptomic studies have significantly improved our understanding of gene expression in various physiological conditions. Therefore, in the current study, we delved into the expression patterns of the *WRKY* TFs in *P. dactylifera* under drought and heat stress conditions. Our investigation unveiled a varied expression profile among *WRKY* genes under these adverse conditions, with many genes exhibiting both up- and down regulations. This indicates that heat and drought stress can potentially trigger the synthesis of stress-protective metabolites crucial for the plant's adaptation to these challenging environmental conditions. For instance, LOC103718774 (PDK_30s65509724g007/*WRKY1*), LOC103704459 (PDK_30s1141221g020/*WRKY57*), LOC103697265 (PDK_30s692531g003/*WRKY49*), LOC103710681 (PDK_30s888801g002/*WRKY2*), LOC103708338 (PDK_30s1093591g002/*WRKY53*), LOC103710422 (PDK_30s839411g021/*WRKY76*), LOC103709750 (PDK_30s663661g001/*WRKY13*), LOC103710925 (PDK_30s1171101g004/*WRKY22*), LOC120112317 (PDK_30s705791g001/*WRKY51*) and LOC103709613 (PDK_30s1067021g003/*WRKY51*), LOC103721507 (PDK_30s742801g002/*WRKY24*), LOC103707065 (PDK_30s716811g001/ *WRKY51*) and LOC103708157 (PDK_30s932191g002/*WRKY41*). Our qRT-PCR results showed that genes LOC103723396 and LOC103718774 were significantly upregulated, suggesting their potential role in stress tolerance. In contrast, the downregulation of the other five genes including LOC103713231, LOC103721327, LOC103721580, LOC120110335, and LOC103707788 indicates that they may be negatively regulated under heat and drought stress conditions. The above analysis suggests that the upregulation of these *PdWRKY* genes during heat and drought treatment could be significant for P. dactylifera in its response to these stresses. However, to thoroughly investigate the potential benefits or negative impacts of these genes on plant responses to various abiotic stressors, further functional investigations are required. The current study lays a solid foundation for future research directions, encompassing functional characterization, evolutionary studies, structural analyses, and pathway investigations. The findings of this study will considerably improve our understanding of the functional roles of *WRKY* genes in date palms and will provide a foundation for future research into plant stress response and adaptability.

## Conclusion

In arid plants, several TFs significantly enhance heat and drought resistance. However, the specific physiological roles of most of these TFs in date palms have not been thoroughly investigated. Therefore, we conducted a genome-wide analysis of *PdWRKY* genes. Phylogenetic analysis identified 9 major clusters of *PdWRKY* genes, and to strengthen our homological analysis, we also constructed a combined phylogenetic tree from *PdWRKYs*, *OsWRKYs*, and *EgWRKYs*. The higher number of orthologous pairs identified in the *WRKY* TFs of *E. guineensis* and *P. dactylifera* indicate that these two taxa share a common ancestor predating the angiosperm taxonomic divergence. The *WRKY* TFs of *P. dactylifera* were analyzed for their gene structure, subcellular localization, conserved motifs, and co-expression networks. These analyses identified several previously uncharacterized genes that have close associations with well-characterized genes. Furthermore, GO and KEGG analysis sheds light on the diverse biological processes in which these genes participate, including transcription regulation, RNA biosynthesis, and response to phytohormones. This study enhances our understanding of *WRKY* TFs in *P. dactylifera* and provides a foundation for further investigations into their roles in heat and drought stress responses, with the aim of improving plant growth and productivity through *WRKY* genes manipulation. Although the results are promising, further functional validation of these genes is required to confirm their roles in stress tolerance and productivity. Our findings open a new window for developing innovative strategies to enhance crop resilience to heat and drought stress. However, due to the limited availability of genomic and transcriptomic data for palm cultivars and the lack of functional validation of *WRKY* TFs under stress conditions, our study predominantly relies on phylogenetic comparisons with *Arabidopsis* and rice. Future research should focus on elucidating the specific physiological functions of these TFs and exploring their potential for improving stress tolerance.

## Supplementary Information

Supplementary Material 1.

Supplementary Material 2.

Supplementary Material 3.

Supplementary Material 4.

Supplementary Material 5.

Supplementary Material 6.

Supplementary Material 7.

Supplementary Material 8.

Supplementary Material 9.

## Data Availability

The data presented in this study can be accessed in the National Center for Biotechnology Information (NCBI) repository under BioProject accession numbers: PRJEB22923 (SRA accession number: ERR2163549-ERR2163580)
