# Supplementary figures and images for: Genome-wide and transcriptome analysis of PdWRKY transcription factors in date palm (Phoenix dactylifera) revealing insights into heat and drought stress tolerance
Source: BMC Genomics. 2025 Jul 1;26:589. doi: 10.1186/s12864-025-11715-6 (PMC12211173; doi:10.1186/s12864-025-11715-6)

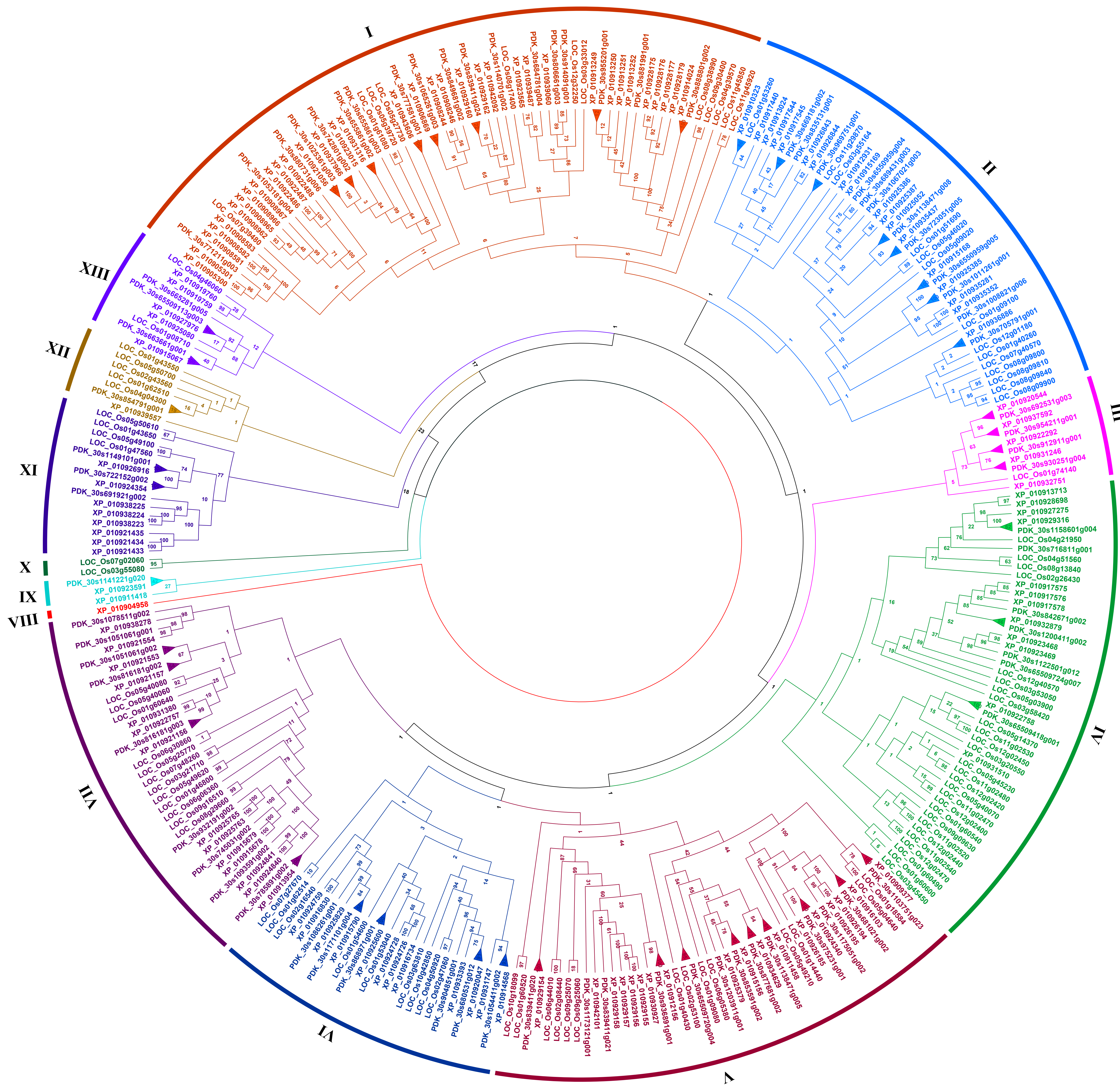

Supplement: Supplementary file 1 — Supplementary Material 1. [file 12864_2025_11715_MOESM1_ESM.pdf]

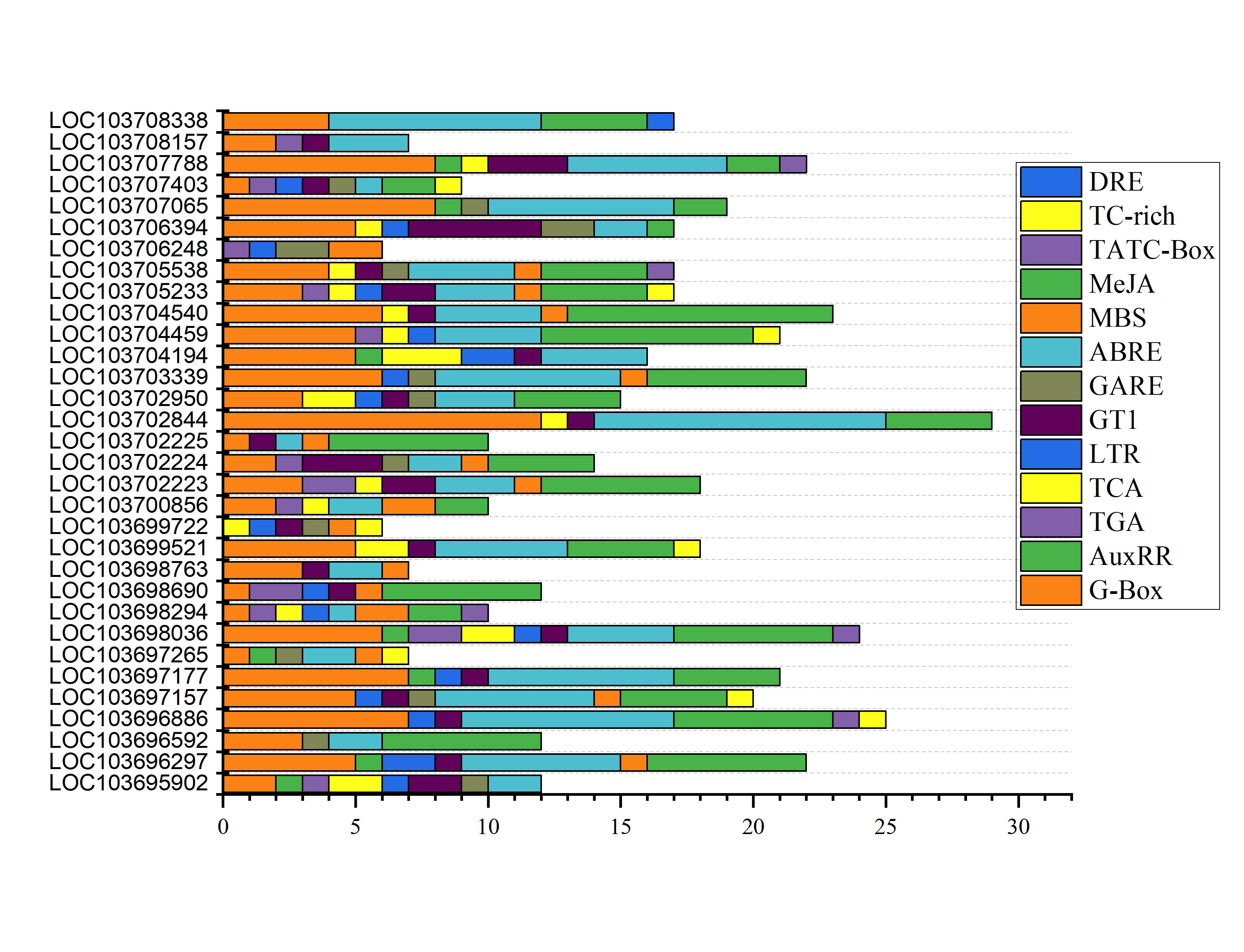

Supplement: Supplementary file 2 — Supplementary Material 2. [file 12864_2025_11715_MOESM2_ESM.png]

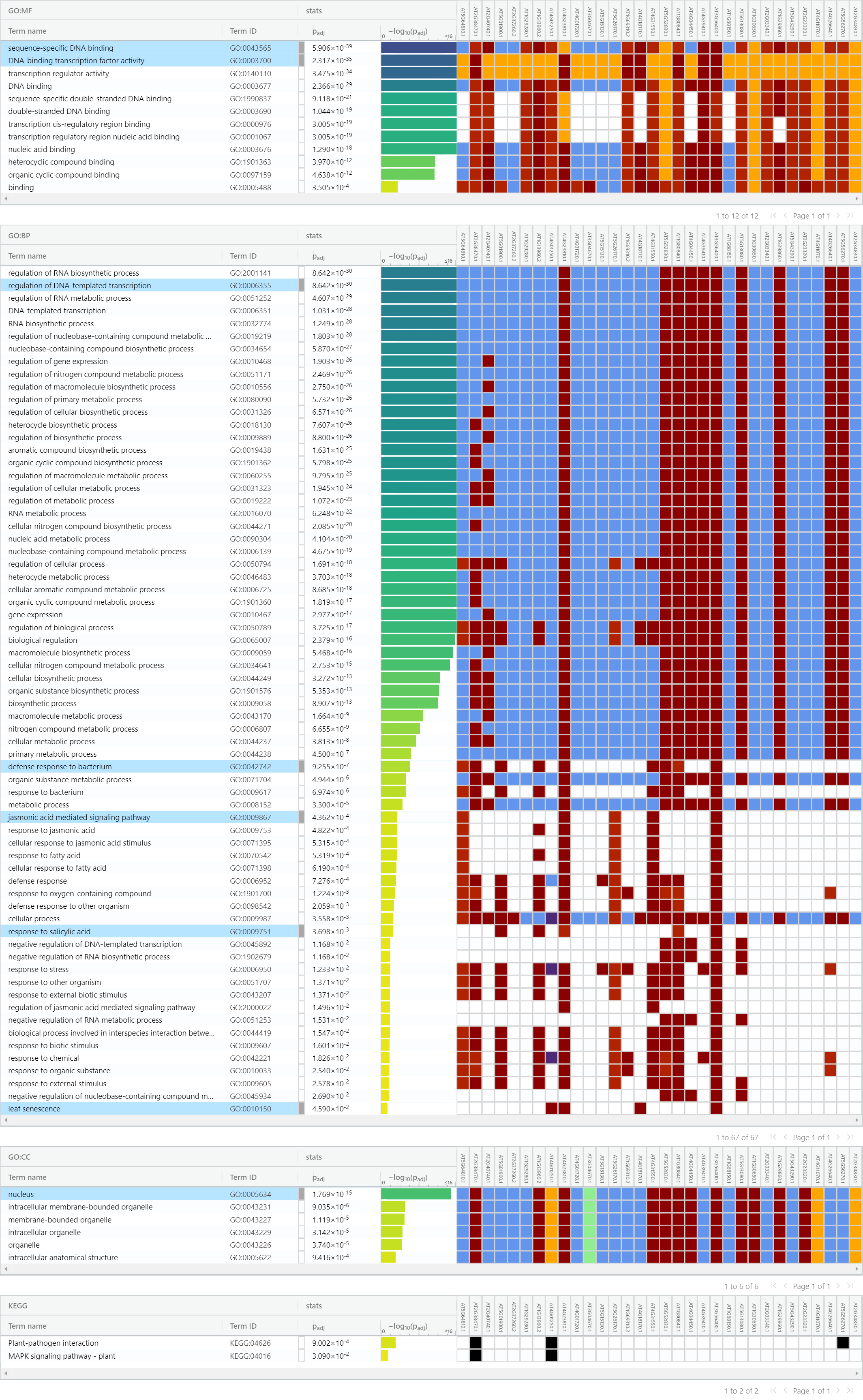

Supplement: Supplementary file 3 — Supplementary Material 3. [file 12864_2025_11715_MOESM3_ESM.png]

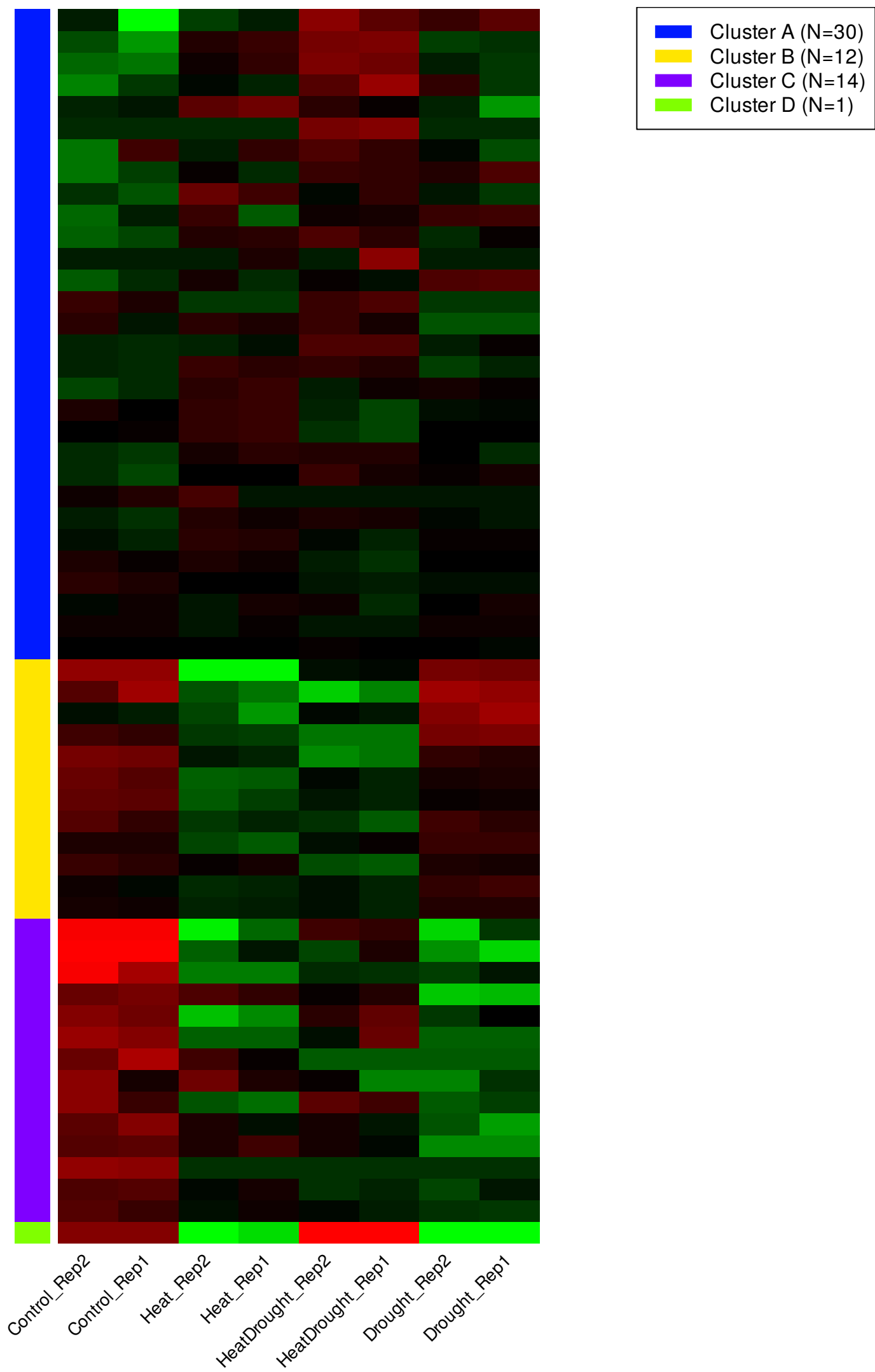

Supplement: Supplementary file 4 — Supplementary Material 4. [file 12864_2025_11715_MOESM4_ESM.pdf]
