# Supplementary material for: Genome-wide and transcriptome analysis of PdWRKY transcription factors in date palm (Phoenix dactylifera) revealing insights into heat and drought stress tolerance
Source: BMC Genomics. 2025 Jul 1;26:589. doi: 10.1186/s12864-025-11715-6 (PMC12211173; doi:10.1186/s12864-025-11715-6)
Supplement: Supplementary file 5 — Supplementary Material 5. [file 12864_2025_11715_MOESM5_ESM.docx]

**Supplementary data**

**Figure S1.** Joined phylogenetic tree constructed from an alignment of 73 *PdWRKYs*, 101 *OsWRKYs* and 131 *EgWRKYs* protein sequences by the ML method with bootstrapping (1,000 replicates) using the MEGA 11 software. The resulting 13 groups are shown in different colors.

**Figure S2.** Cis-regulatory elements identified in the 2000 bp upstream region of *P. dactylifera* *WRKY* genes, associated with diverse functions.

**Figure S3.** The Enrichment plot of GO enrichment and KEGG pathway analysis. The color intensity of the nodes represents the count and-log10(q) values of pathways, respectively.

**Figure S4.** Clustering of *PdWRKY* genes into four groups to identify potential co-functioning genes in various stress-responsive pathways.

**Table S1.** iTAK and LOC accession numbers of *PdWRKYs*.

**Table S2.** Sequences of primers used in qRT-PCR.

**Table S3.** Diverse cis-elements in the *P. dactylifera* *WRKY* gene family.

**Table S4.** Standard and given names of the chromosomes in figure 6 and their lengths.
